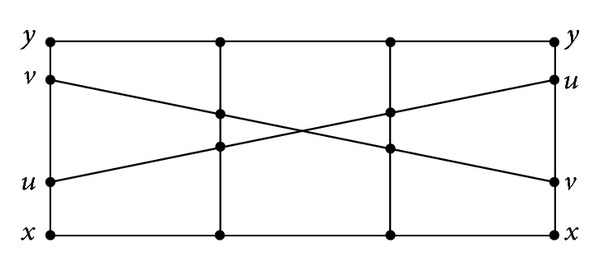# *m*-Polar Fuzzy Sets: An Extension of Bipolar Fuzzy Sets

**DOI:** 10.1155/2014/416530

**Published:** 2014-06-12

**Authors:** Juanjuan Chen, Shenggang Li, Shengquan Ma, Xueping Wang

**Affiliations:** ^1^College of Mathematics and Information Science, Shaanxi Normal University, Xi'an 710062, China; ^2^School of Sciences, Xi'an University of Technology, Xi'an 710056, China; ^3^College of Mathematics and Software Science, Sichuan Normal University, Chengdu 610066, China

## Abstract

Recently, bipolar fuzzy sets have been studied and applied a bit enthusiastically and a bit increasingly. In this paper we prove that bipolar fuzzy sets and [0,1]^2^-sets (which have been deeply studied) are actually cryptomorphic mathematical notions. Since researches or modelings on real world problems often involve multi-agent, multi-attribute, multi-object, multi-index, multi-polar information, uncertainty, or/and limit process, we put forward (or highlight) the notion of *m*-polar fuzzy set (actually, [0,1]^*m*^-set which can be seen as a generalization of bipolar fuzzy set, where *m* is an arbitrary ordinal number) and illustrate how many concepts have been defined based on bipolar fuzzy sets and many results which are related to these concepts can be generalized to the case of *m*-polar fuzzy sets. We also give examples to show how to apply *m*-polar fuzzy sets in real world problems.

## 1. Introduction and Preliminaries

Set theory and logic systems are strongly coupled in the development of modern logic. Classical logic corresponds to the crisp set theory, and fuzzy logic is associated with fuzzy set theory which was proposed by Zadeh in his pioneer work [1].

**Definition 1 .**

An *L*-subset (or an *L*-set) on the set *X* is a synonym of a mapping *A* : *X* → *L*, where *L* is a lattice (cf. [2]). When *L* = [0,1] (the ordinary closed unit interval with the ordinary order relation), an *L*-set on *X* will be called a fuzzy set on *X* (cf. [1]).

The theory of fuzzy sets has become a vigorous area of research in different disciplines including medical and life sciences, management sciences, social sciences, engineering, statistics, graph theory, artificial intelligence, pattern recognition, robotics, computer networks, decision making, and automata theory.

An extension of fuzzy set, called bipolar fuzzy set, was given by Zhang [3] in 1994.

**Definition 2 (see Zhang [3]).**

A bipolar fuzzy set is a pair (*μ* ^+^, *μ* ^−^), where *μ* ^+^ : *X* → [0,1] and *μ* ^−^ : *X* → [−1,0] are any mappings. The set of all bipolar fuzzy sets on *X* is denoted by BF(*X*).

Bipolar fuzzy sets are an extension of fuzzy sets whose membership degree range is [−1,1]. In a bipolar fuzzy set, the membership degree 0 of an element means that the element is irrelevant to the corresponding property, the membership degree (0,1] of an element indicates that the element somewhat satisfies the property, and the membership degree [−1,0) of an element indicates that the element somewhat satisfies the implicit counter-property. The idea which lies behind such description is connected with the existence of “bipolar information” (e.g., positive information and negative information) about the given set. Positive information represents what is granted to be possible, while negative information represents what is considered to be impossible. Actually, a wide variety of human decision making is based on double-sided or bipolar judgmental thinking on a positive side and a negative side. For instance, cooperation and competition, friendship and hostility, common interests and conflict of interests, effect and side effect, likelihood and unlikelihood, feedforward and feedback, and so forth are often the two sides in decision and coordination. In the traditional Chinese medicine (TCM for short), “yin” and “yang” are the two sides. Yin is the feminine or negative side of a system and yang is the masculine or positive side of a system. The coexistence, equilibrium, and harmony of the two sides are considered a key for the mental and physical health of a person as well as for the stability and prosperity of a social system. Thus bipolar fuzzy sets indeed have potential impacts on many fields, including artificial intelligence, computer science, information science, cognitive science, decision science, management science, economics, neural science, quantum computing, medical science, and social science (cf. [4–45]). In recent years bipolar fuzzy sets seem to have been studied and applied a bit enthusiastically and a bit increasingly (cf. [4–45]). This is the chief motivation for us to introduce and study *m*-polar fuzzy sets.

The first object of this note is to answer the following question on bipolar fuzzy sets.

**Question 1 .**

Is bipolar fuzzy set a very intuitive *L*-set?

The answer to Question 1 is positive. We will prove in this note that there is a natural one-to-one correspondence between BF(*X*) and 2(*X*) (for the set of all [0,1]^2^-sets on *X*, see Theorem 5) which preserves all involved properties. This makes the notion of bipolar fuzzy set more intuitive. Since properties of *L*-sets have already been studied very deeply and exhaustively, this one-to-one correspondence may be beneficial for both researchers interested in above-mentioned papers and related fields (because they can use these properties directly and even cooperate with theoretical fuzzy mathematicians for a possible higher-level research) and theoretical fuzzy mathematicians as well (because cooperation with applied fuzzy mathematicians and practitioners probably makes their research more useful).

We notice that “multipolar information” (not just bipolar information which corresponds to two-valued logic) exists because data for a real world problem are sometimes from *n* agents (*n* ≥ 2). For example, the exact degree of telecommunication safety of mankind is a point in [0,1]^*n*^ (*n* ≈ 7 × 10^9^) because different person has been monitored different times. There are many other examples: truth degrees of a logic formula which are based on *n* logic implication operators (*n* ≥ 2), similarity degrees of two logic formulas which are based on *n* logic implication operators (*n* ≥ 2), ordering results of a magazine, ordering results of a university, and inclusion degrees (resp., accuracy measures, rough measures, approximation qualities, fuzziness measures, and decision performance evaluations) of a rough set. Thus our second object of this note is to answer the following question on extensions of bipolar fuzzy sets.

**Question 2 .**

How to generalize bipolar fuzzy sets to multipolar fuzzy sets and how to generalize results on bipolar fuzzy sets to the case of multipolar fuzzy sets?

The idea to answer Question 2 is from the answer to Question 1, intuitiveness of the point-wise order on [0,1]^*m*^ (see Remark 3), and the proven corresponding results on bipolar fuzzy sets. We put forward the notion of *m*-polar fuzzy set (an extension of bipolar fuzzy set) and point out that many concepts which have been defined based on bipolar fuzzy sets and many results related to these concepts can be generalized to the case of *m*-polar fuzzy sets (see Remarks 7 and 8 for details).

Apart from the backgrounds (e.g., “multipolar information”) of *m*-polar fuzzy sets, the following question on further applications (particularly, further applications in real world problems) of *m*-polar fuzzy sets should also be considered.

**Question 3 .**

How to find further possible applications of *m*-polar fuzzy sets in real world problems?

Question 3 can be answered as in the case of bipolar fuzzy sets since researches or modelings on real world problems often involve multiagent, multiattribute, multiobject, multi-index, multipolar information, uncertainty, or/and limits process. We will give examples to demonstrate it (see Examples 9–14).

**Remark 3 .**

In this note [0,1]^*m*^ (*m*-power of [0,1]) is considered a poset with the point-wise order ≤, where *m* is an arbitrary ordinal number (we make an appointment that *m* = {*n*∣*n* < *m*} when *m* > 0), ≤ (which is actually very intuitive as illustrated below) is defined by *x* ≤ *y*⇔*p*_*i*_(*x*) ≤ *p*_*i*_(*y*) for each *i* ∈ *m* (*x*, *y* ∈ [0,1]^*m*^), and *p*_*i*_ : [0,1]^*m*^ → [0,1] is the *i*th projection mapping (*i* ∈ *m*).

**Figure 1:**
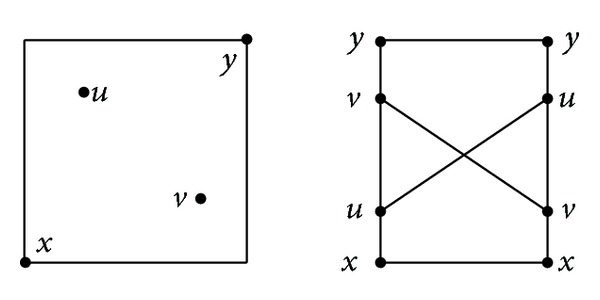


1. When *m* = 2, [0,1]^2^ is the ordinary closed unit square in Euclidean plane *R* ^2^. The righter (resp., the upper) a point in this square is, the larger it is. Let *x* = 〈0,0〉 = 0 (the smallest element of [0,1]^2^), *u* = 〈0.25,0.75〉, *v* = 〈0.75,0.25〉, and *y* = 〈1,1〉 (the largest element of [0,1]^2^). Then *x* ≤ *z* ≤ *y* for all *z* ∈ [0,1]^2^ (especially, *x* ≤ *u* ≤ *y* and *x* ≤ *v* ≤ *y* hold). Notice that *u*≰*v*≰*u* because both *p*_0_(*u*) = 0.25 ≤ 0.75 = *p*_0_(*v*) and *p*_1_(*u*) = 0.75 ≥ 0.25 = *p*_1_(*v*) hold. The order relation ≤ on [0,1]^2^ can be illustrated in at least two ways (see Figure 1).
2. When *m* > 2, the order relation ≤ on [0,1]^*m*^ can be illustrated in at least one way (see Figure 2 for the case *m* = 4, where *x* ≤ *u* ≤ *y*, *x* ≤ *v* ≤ *y*).

## 2. Main Results

In this section we will prove that a bipolar fuzzy set is just a very specific *L*-set, that is, [0,1]^2^-set. We also put forward (or highlight) the notion of *m*-polar fuzzy set (which is still a special *L*-set, i.e., [0,1]^*m*^-set, although it is a generalization of bipolar fuzzy set) and point out that many concepts which have been defined based on bipolar fuzzy sets and results related to these concepts can be generalized to the case of *m*-polar fuzzy sets.

**Definition 4 .**

An *m*-polar fuzzy set (or a [0,1]^*m*^-set) on *X* is exactly a mapping *A* : *X* → [0,1]^*m*^. The set of all *m*-polar fuzzy sets on *X* is denoted by *m*(*X*).

The following theorem shows that bipolar fuzzy sets and 2-polar fuzzy sets are cryptomorphic mathematical notions and that we can obtain concisely one from the corresponding one.

**Theorem 5 .**

Let *X* be a set. For each bipolar fuzzy set (*μ* ^+^, *μ* ^−^) on *X*, define a 2-polar fuzzy set

(1) $$\begin{array}{c} \varphi \left(\mu^{+},\mu^{-}\right)=A_{\mu }:X⟶{\left[0,1\right]}^{2} \end{array}$$

on *X* by putting

(2) $$\begin{array}{c} A_{\mu }\left(x\right)=\langle \mu^{+}\left(x\right),-\mu^{-}\left(x\right)\rangle \;\left(\forall x\in X\right). \end{array}$$

Then we obtain a one-to-one correspondence

(3) $$\begin{array}{c} \varphi :BF\left(X\right)⟶2\left(X\right); \end{array}$$

its inverse mapping *ψ* : 2(*X*) → *BF*(*X*) is given by *ψ*(*A*) = (*μ*_*A*_^+^, *μ*_*A*_^−^)  (∀*A* ∈ 2(*X*)), *μ*_*A*_^+^(*x*) = *p*_0_∘*A*(*x*)  (∀*x* ∈ *X*), and *μ*_*A*_^−^(*x*) = −*p*_1_∘*A*(*x*)  (∀*x* ∈ *X*).

**Proof**

Obviously, both *φ* and *ψ* are mappings. For each (*μ* ^+^, *μ* ^−^)∈ BF(*X*),

(4) [ψ∘φ(μ+,μ−)](x) =〈p0∘φ(μ+,μ−)(x),−p1∘φ(μ+,μ−)(x)〉 =〈p0(〈μ+(x),μ−(x)〉),−p1(〈μ+(x),μ−(x)〉)〉 =〈μ+(x),−μ−(x)〉=(μ+,μ−)(x) (∀x∈X),

which means [*ψ*∘*φ*(*μ* ^+^, *μ* ^−^)] = (*μ* ^+^, *μ* ^−^). Again, for each *A* ∈ 2(*X*) and each *x* ∈ *X*,

(5) [φ∘ψ(A)](x)=φ(μA+,μA−)(x)=〈μA+(x),−μA−(x)〉=A(x),

which means *φ*∘*ψ*(*A*) = *A*.

**Example 6 .**

Let (*μ* ^+^, *μ* ^−^) be a bipolar fuzzy set, where *X* = {*u*, *v*, *w*, *x*, *y*, *z*} is a six-element set and *μ* ^+^ : *X* → [0,1] and *μ* ^−^ : *X* → [−1,0] are defined by

(6) $$\begin{array}{c} \mu^{+}=\left\{\frac{0.4}{u},\frac{0.5}{v},\frac{0.3}{w},\frac{1}{x},\frac{1}{y},\frac{0.6}{z}\right\},\\ \mu^{-}=\left\{\frac{-0.3}{u},\frac{-0.6}{v},\frac{-1}{w},\frac{-0.2}{x},\frac{-1}{y},\frac{-0.5}{z}\right\}. \end{array}$$

Then the corresponding 2-polar fuzzy set on *X* is

(7) Aμ={〈0.4,0.3〉u,〈0.5,0.6〉v,〈0.3,1〉w,〈1,0.2〉x,〈1,1〉y,〈0.6,0.5〉z}.

In the rest of this note, we investigate the possible applications of *m*-polar fuzzy sets. First we consider the theoretic applications of *m*-polar fuzzy sets. More precisely, we will give some remarks to illustrate how many concepts which have been defined based on bipolar fuzzy sets and results related to these concepts can be generalized to the case of *m*-polar fuzzy sets (see the following Remarks 7 and 8).

**Remark 7 .**

The notions of bipolar fuzzy graph (see [4, 45]) and fuzzy graph (see [46, 47]) can be generalized to the convenient (because it allows a computing in computers) and intuitive notion of *m*-polar fuzzy graph. An *m*-polar fuzzy graph with an underlying pair (*V*, *E*) (where *E*⊆*V* × *V* is symmetric; i.e., it satisfies 〈*x*, *y*〉∈*E*⇔〈*y*, *x*〉∈*E*) is defined to be a pair *G* = (*A*, *B*), where *A* : *V* → [0,1]^*m*^ (i.e., an *m*-polar fuzzy set on *V*) and *B* : *E* → [0,1]^*m*^ (i.e., an *m*-polar fuzzy set on *E*) satisfy *B*(〈*x*, *y*〉) ≤ inf⁡{*A*(*x*), *A*(*y*)}  (∀〈*x*, *y*〉∈*E*); *A* is called the *m*-polar fuzzy vertex set of *V* and *B* is called the *m*-polar fuzzy edge set of *E*. An *m*-polar fuzzy graph *G* = (*A*, *B*) with an underlying pair (*V*, *E*) and satisfying *B*(〈*x*, *y*〉) = *B*(〈*y*, *x*〉)  (∀〈*x*, *y*〉∈*E*) and *B*(〈*x*, *x*〉) = 0  (∀*x* ∈ *V*) is called a simple *m*-polar fuzzy graph, where 0 is the smallest element of [0,1]^*m*^. An *m*-polar fuzzy graph *G* = (*A*, *B*) with an underlying pair (*V*, *E*) and satisfying *B*(〈*x*, *y*〉) = inf⁡{*A*(*x*), *A*(*y*)}  (∀〈*x*, *y*〉∈*E*) is called a strong *m*-polar fuzzy graph. The complement of a strong *m*-polar fuzzy graph *G* = (*A*, *B*) (which has an underlying pair (*V*, *E*)) is a strong *m*-polar fuzzy graph $\bar{G}=(A,\bar{B})$ with an underlying pair (*V*, *E*), where $\bar{B}:E\to {\left[0,1\right]}^{m}$ is defined by (〈*x*, *y*〉∈*E*, *i* ∈ *m*)

(8) pi∘B¯(〈x,y〉) ={0,pi∘B(〈x,y〉)>0,inf⁡{pi∘A(x),pi∘A(y)},pi∘B(〈x,y〉)=0.

Give two *m*-polar fuzzy graphs (with underlying pairs (*V*_1_, *E*_1_) and (*V*_2_, *E*_2_), resp.) *G*_1_ = (*A*_1_, *B*_1_) and *G*_2_ = (*A*_2_, *B*_2_). A homomorphism from *G*_1_ to *G*_2_ is a mapping *f* : *V*_1_ → *V*_2_ which satisfies *A*_1_(*x*) ≤ *A*_2_(*f*(*x*))  (∀*x* ∈ *V*_1_) and *B*_1_(〈*x*, *y*〉) ≤ *B*_2_(〈*f*(*x*), *f*(*y*)〉)  (∀〈*x*, *y*〉∈*E*_1_). An isomorphism from *G*_1_ to *G*_2_ is a bijective mapping *f* : *V*_1_ → *V*_2_ which satisfies *A*_1_(*x*) = *A*_2_(*f*(*x*))  (∀*x* ∈ *V*_1_) and *B*_1_(〈*x*, *y*〉) = *B*_2_(〈*f*(*x*), *f*(*y*)〉)  (∀〈*x*, *y*〉∈*E*_1_). A weak isomorphism from *G*_1_ to *G*_2_ is a bijective mapping *f* : *V*_1_ → *V*_2_ which is a homomorphism and satisfies *A*_1_(*x*) = *A*_2_(*f*(*x*))  (∀*x* ∈ *V*_1_). A strong *m*-polar fuzzy graph *G* is called self-complementary if $G≃\bar{G}$ (i.e., there exists an isomorphism between *G* and its complement $\bar{G}$).

It is not difficult to verify the following conclusions (some of which generalize the corresponding results in [1, 45]).

(1) In a self-complementary strong *m*-polar fuzzy graph *G* = (*A*, *B*) (with an underlying pair (*V*, *E*)), we have
  (9) pi∘B¯(〈x,y〉) =inf⁡⁡{pi∘A(x),pi∘A(y)}−pi∘B(〈x,y〉)(i∈m,〈x,y〉∈E),∑x≠ypi∘B(〈x,y〉) =12∑x≠yinf⁡{pi∘A(x),pi∘A(y)} (i∈m).
(2) A strong *m*-polar fuzzy graph *G* = (*A*, *B*) (with an underlying pair (*V*, *E*)) is self-complementary if and only if it satisfies
  (10) pi∘B(〈x,y〉)=12inf⁡{pi∘A(x),pi∘A(y)}(∀i∈m,∀〈x,y〉∈E).
(3) If *G*_1_ and *G*_2_ are strong *m*-polar fuzzy graphs, then *G*_1_≃*G*_2_ if and only if $\bar{G_{1}}≃\bar{G_{2}}$.
(4) Let *G*_1_ and *G*_2_ be strong *m*-polar fuzzy graphs. If there is a weak isomorphism from *G*_1_ to *G*_2_, then there is a weak isomorphism from $\bar{G_{2}}$ to $\bar{G_{1}}$.

**Remark 8 .**

The fuzzifications or bipolar fuzzifications of some algebraic concepts (such as group, *K*-algebra, incline algebra (cf. [48]), ideal, filter, and finite state machine) can be generalized to the case of *m*-polar fuzzy sets. An *m*-polar fuzzy set *A* : *G* → [0,1]^*m*^ is called an *m*-polar fuzzy subgroup of a group (*G*, ∘) if it satisfies *A*(*x*∘*y* ^−1^) ≥ inf⁡{*A*(*x*), *A*(*y*)}  (∀*x*, *y* ∈ *G*). An *m*-polar fuzzy set *A* : *G* → [0,1]^*m*^ is called an *m*-polar fuzzy subalgebra of a *K*-algebra (*G*, ∘, *e*, ⊙) if it satisfies *A*(*x*∘*y*) ≥ inf⁡{*A*(*x*), *A*(*y*)}  (∀*x*, *y* ∈ *G*). An *m*-polar fuzzy set *A* : *X* → [0,1]^*m*^ is called an *m*-polar fuzzy subincline of an incline (*X*, +, ∗) if it satisfies (*x*∗*y*) ≥ inf⁡{*A*(*x*), *A*(*y*)}  (∀*x*, *y*∈*G*); it is called an *m*-polar fuzzy ideal (resp., an *m*-polar fuzzy filter) of (*X*, +, ∗) if it is an *m*-polar fuzzy subincline of (*X*, +, ∗) and satisfies *A*(*x*) ≥ *A*(*y*) whenever *x* ≤ *y* (resp., satisfies *A*(*x*) ≤ *A*(*y*) whenever *x* ≤ *y*). An *m*-polar fuzzy finite state machine is a triple *M* = (*Q*, *X*, *A*), where *Q* and *X* are finite nonempty sets (called the set of states and the set of input symbols, resp.) and *A* : *Q* × *X* × *Q* → [0,1]^*m*^ is any *m*-polar fuzzy set on *Q* × *X* × *Q*. Moreover, if *B* : *Q* → [0,1]^*m*^ is an *m*-polar fuzzy set on *Q* satisfying

(11) B(q)≥inf⁡{B(p),A(〈p,x,q〉)}(∀〈p,x,q〉∈Q×X×Q),

then *M*_0_ = (*Q*, *X*, *A*, *B*) is called an *m*-polar subsystem of *M*. Furthermore, let X* be the set of all words of elements of *X* of finite length and *λ* be the empty word in X* (cf. [28]). Then one can define a *m*-polar fuzzy set A* : *Q* × X* × *Q* → [0,1]^*m*^ on *Q* × X* × *Q* by putting

(12) A∗(〈q,λ,p〉)={1,if q=p,0,if q≠p,A∗(〈q,x,x,p〉)=sup⁡r∈Q{A∗(〈q,x,r〉),A∗(〈r,x,p〉)}(∀〈q,x,x,p〉∈Q×X∗×X×Q),

where 1 is the biggest element of [0,1]^*m*^.

The following conclusions hold.

1. An *m*-polar fuzzy set *A* : *G* → [0,1]^*m*^ is an *m*-polar fuzzy subgroup of a group (*G*, ∘) if and only if *A*_[*a*]_ = {*x* ∈ *G*∣*A*(*x*) ≥ *a*} is *∅* or *A*_[*a*]_ is a subgroup of (*G*, ∘)  (∀*a* ∈ [0,1]^*m*^).
2. An *m*-polar fuzzy set *A* : *G* → [0,1]^*m*^ is an *m*-polar fuzzy subalgebra of a *K*-algebra (*G*, ∘, *e*, ⊙) if and only if *A*_[*a*]_ = {*x* ∈ *G*∣*A*(*x*) ≥ *a*} is *∅* or *A*_[*a*]_ is a subalgebra of (*G*, ∘, *e*, ⊙)  (∀*a* ∈ [0,1]^*m*^).
3. An *m*-polar fuzzy set *A* : *X* → [0,1]^*m*^ is an *m*-polar fuzzy subincline (resp., an *m*-polar fuzzy ideal, an *m*-polar fuzzy filter) of an incline (*X*, +, ∗) if and only if *A*_[*a*]_ is a subincline (resp., ideal, filter) of (*X*, +, ∗)  (∀*a* ∈ [0,1]^*m*^).
4. Let *M* = (*Q*, *X*, *A*) be an *m*-polar fuzzy finite state machine and *B* : *Q* → [0,1]^*m*^ be an *m*-polar fuzzy set on *Q*. Then (*Q*, *X*, *A*, *B*) is an *m*-polar subsystem of *M* if and only if *B*(*q*) ≥ inf⁡{*B*(*p*), A*(〈*p*, **x**, *q*〉)}  (∀〈*p*, **x**, *q*〉∈*Q* × X* × *Q*). Please see [49, 50] for more results.

Next we consider the applications of *m*-polar fuzzy sets in real world problems.

**Example 9 .**

Let *X* be a set consisting of five patients *x*, *y*, *z*, *u*, and *v* (thus *X* = {*x*, *y*, *z*, *u*, *v*}). They have diagnosis data consisting of three aspects, diagnosis datum of *x* is (*x*) = 〈0.49,0.46,0.51〉, where datum 0.5 represents “normal” or “OK.” Suppose *A*(*y*) = 〈0.45,0.42,0.59〉, *A*(*z*) = 〈0.50,0.40,0.54〉, *A*(*u*) = 〈0.40,0.49,0.60〉, and *A*(*v*) = 〈0.51,0.52,0.50〉. Then we obtain a 3-polar fuzzy set *A* : *X* → [0,1]^3^ which can describe the situation; this 3-polar fuzzy set can also be written as follows:

(13) A={〈0.49,0.46,0.51〉x,〈0.45,0.42,0.59〉y,〈0.50,0.40,0.54〉z,〈0.40,0.49,0.60〉u,〈0.51,0.52,0.50〉v}.

**Example 10 .**

*m*-polar fuzzy sets can be used in decision making. In many decision making situations, it is necessary to gather the group consensus. This happens when a group of friends decides which movie to watch, when a company decides which product design to manufacture, and when a democratic country elects its leaders. For instance, we consider here only the case of election. Let *X* = {*x*, *y*, *z*,…, *u*, *v*} be the set of voters and *C* = {*c*_1_, *c*_2_, *c*_3_, *c*_4_} be the set of all the four candidates. Suppose the voting is weighted. For each candidate *c* ∈ *C*, a voter in {*x*, *y*, *z*} can send a value in [0,1] to *c*, but a voter in *X* − {*x*, *y*, *z*} can only send a value in [0.1,0.8] to *c*. Suppose *A*(*x*) = 〈0.9,0.4,0.01,0.1〉 (which means the preference degrees of *x* corresponding to *c*_1_, *c*_2_, *c*_3_, and *c*_4_ are 0.9, 0.4, 0.01, and 0.1, resp.), *A*(*y*) = 〈0.2,0.3,0.8,0.1〉, *A*(*z*) = 〈0.8,0.9,0.8,0.2〉,…, *A*(*u*) = 〈0.6,0.8,0.8,0.1〉, and *A*(*v*) = 〈0.7,0.8,0.4,0.2〉. Then we obtain a 4-polar fuzzy set *A* : *X* → [0,1]^4^ which can describe the situation; this 4-polar fuzzy set can also be written as follows:

(14) A={〈0.9,0.4,0.01,0.1〉x,〈0.2,0.3,0.8,0.1〉y,〈0.8,0.9,0.8,0.2〉z,…,〈0.6,0.8,0.8,0.1〉u,〈0.7,0.8,0.4,0.2〉v}.

**Example 11 .**

*m*-polar fuzzy sets can be used in cooperative games (cf. [51]). Let *X* = {*x*_1_, *x*_2_,…, *x*_*n*_} be the set of *n* agents or players (*n* ≥ 1), *m* = {0,1,…, *m* − 1} be the set of the grand coalitions, and *A* : *X* → [0,1]^*m*^ be an *m*-polar fuzzy set, where *p*_*i*_∘*A*(*x*) is the degree of player *x* participating in coalition *i* (*x* ∈ *X*, *i* ∈ *m*). Again let *v* : [0,1]^*m*^ → *R* (the set of all real numbers) be a mapping satisfying *v*(0) = 0. Then the mapping *v*∘*A* : *X* → *R* is called a cooperative game, where *v*∘*A*(*x*) represents the amount of money obtained by player *x* under the coalition participating ability *A*(*x*) (*x* ∈ *X*).

(1) (a public good game; compare with [51, Example 6.5]) Suppose *n* agents *x*_1_, *x*_2_,…, *x*_*n*_ want to create a facility for joint use. The cost of the facility depends on the sum of the participation levels (or degrees) of the agents and it is described by

(15) $$\begin{array}{c} k\left(\overset{n}{\underset{i=1}{\sum }}B\left(x_{i}\right)\right), \end{array}$$

where *k* : [0, *n*] → *R* is a continuous monotonic increasing function with *k*(0) = 0 and *B* : *X* → [0,1] is a mapping. Let *A* : *X* = {*x*_1_, *x*_2_,…, *x*_*n*_}→[0,1]^*n*^ be a mapping satisfying *p*_*j*_∘*A*(*x*_*i*_) = *B*(*x*_*i*_) (if *j* = *i*) or 0 (otherwise) (*i* = 1,2,…, *n*). Then a cooperative game model *v* ∘*A* : *X* → *R* is established, where *v* : [0,1]^*n*^ → *R* is defined by

(16) v(〈s1,s2,…,sn〉)=∑i=1ngi(si)−1nk(∑i=1nB(xi))(∀〈s1,s2,…,sn〉∈[0,1]n),

and the function *g*_*i*_ : [0,1] → *R* is continuously monotonic increasing with *g*_*i*_(0) = 0 (*i* = 1,2,…, *n*). Obviously, the gain of agent *x*_*i*_ (with participation level *B*(*x*_*i*_)) is

(17) $$\begin{array}{c} v\circ A\left(x_{i}\right)=g_{i}\circ B\left(x_{i}\right)-\frac{1}{n}k\left(\overset{n}{\underset{i=1}{\sum }}B\left(x_{i}\right)\right), \end{array}$$

and the total gain is

(18) $$\begin{array}{c} \overset{n}{\underset{i=1}{\sum }}v\circ A\left(x_{i}\right)=\overset{n}{\underset{i=1}{\sum }}g_{i}\circ B\left(x_{i}\right)-k\left(\overset{n}{\underset{i=1}{\sum }}B\left(x_{i}\right)\right). \end{array}$$

(2) There are two goods, denoted *g*_1_ and *g*_2_, and three agents *a*, *b*, and *c* with endowments (*ε*, *ε*), (1 − *ε*, 0), and (0,1 − *ε*)  (0 < *ε* ≤ 1). Let *v* : [0,1]^2^ → *R* be any mapping satisfying *v*(〈0,0〉) = 0. Then the corresponding cooperative game model is *v*∘*A* : *X* = {*a*, *b*, *c*} → *R*, where

(19) $$\begin{array}{c} A=\left\{\frac{\langle \varepsilon ,\varepsilon \rangle }{a},\frac{\langle 1-\varepsilon ,0\rangle }{b},\frac{\langle 0,1-\varepsilon \rangle }{c}\right\},\\ v\circ A=\left\{\frac{v\left(\langle \varepsilon ,\varepsilon \rangle \right)}{a},\frac{v\left(\langle 1-\varepsilon ,0\rangle \right)}{b},\frac{v\left(\langle 0,1-\varepsilon \rangle \right)}{c}\right\}. \end{array}$$

**Example 12 .**

*m*-polar fuzzy sets can be used to define weighted games. A weighted game is a 4-tuple (*X*, *P*, *W*, Δ), where *X* = {*x*_1_, *x*_2_,…, *x*_*n*_} is the set of *n* players or voters (*n* ≥ 2), *P* is a collection of fuzzy sets on *X* (called coalitions) such that (*P*, ≤) is upper set (i.e., a fuzzy set *Q* on *X* belongs to *P* if *Q* ≥ *P* for some *P* ∈ *P*), *W* : *X* → [0,1]^*m*^ is an *m*-polar fuzzy set on *X* (called voting weights), and Δ⊆[0, +*∞*)^*m*^ − {0} (called quotas). Imagine a situation: three people, *x*, *y*, and *z*, vote for a proposal on releasing of a student. Suppose that *x* casts 200 US Dollars and lose 80 hairs on her head votes each, *y* casts 60000 US Dollars and 100 grams* Cordyceps sinensis* votes each, *z* casts 100000 US Dollars and 100 grams gold votes each. Then an associated weighted game model is (*X*, *P*, *W*, Δ), where *X* = {*x*, *y*, *z*} and *P* is a collection of fuzzy sets on *X* with (*P*, ≤) an upper set, *m* = 4,

(20) W={〈200/160200,80/80,0,0〉x,〈60000/160200,0,100/100,0〉y,〈100000/160200,0,0,100/100〉z},Δ={〈200000,0,0,0〉,〈100000,300,0,0〉,〈0,0,500,0〉,〈0,0,0,65000〉}.

(1) If the situation is a little simple, *x* casts [100,300]  US Dollars (i.e., the cast is between 100 US Dollars and 300 US Dollars, where [100,300] is an interval number which can be looked as a point [0, +*∞*)^2^) votes each, *y* casts [50000,70000]  US Dollars votes each, *z* casts [90000,110000]  US Dollars votes each, and quota is [100000,120000]. Then the corresponding weighted game model is (*X*, *P*, *W*, [100000,120000]), where *P* is a collection of fuzzy sets on *X* with (*P*, ≤) an upper set, *m* = 1, and

(21) $$\begin{array}{c} W=\left\{\frac{400/320400}{x},\frac{120000/320400}{y},\frac{200000/320400}{z}\right\}. \end{array}$$

(2) If the situation is more simple, *x* casts 200 US Dollars votes each, *y* casts 60000 US Dollars votes each, *z* casts 100000 US Dollars votes each, and quota is 110000. Then the corresponding weighted game model is (*X*, *P*, *W*, 110000), where *P* = {{*x*, *z*}, {*y*, *z*}, {*x*, *y*, *z*}}, *m* = 1, and

(22) $$\begin{array}{c} W=\left\{\frac{200/160200}{x},\frac{60000/160200}{y},\frac{100000/160200}{z}\right\}. \end{array}$$

Notice that the subset {*x*, *z*}⊆*X* is exactly a fuzzy set *A* : *X* → [0,1] on *X* defined by *A*(*x*) = *A*(*z*) = 1 and *A*(*y*) = 0.

**Example 13 .**

*m*-polar fuzzy sets can be used as a model for clustering or classification. Consider a set *X* consisting of *n* students *x*_1_, *x*_2_,…, *x*_*n*_  (*n* ≥ 2) in Chinese middle school. For a student *x* ∈ *X*, we use integers *x*_1_ (resp., *x*_2_,…, *x*_6_) in [0,100] to denote the average score of Mathematics (resp., Physics, Chemistry, Biology, Chinese, and English), and

(23) A(x)=〈x1×0.01,x2×0.01,x3×0.01,x4×0.01,x5×0.01,x6×0.01〉.

Then we obtain a 6-polar fuzzy set model *A* : *X* → [0,1]^6^, which can be used for clustering or classification of these students.

**Example 14 .**

*m*-polar fuzzy sets can be used to define multivalued relations.

1. Consider a set *X* consisting of *n* net users (resp., patients) *x*_1_, *x*_2_,…, *x*_*n*_  (*n* ≥ 2). For net users (resp., patients) *x*, *y* ∈ *X*, we use (*x*, *y*, *j*) to denote the similarity between *x* and *y* in *j*th aspect (1 ≤ *j* ≤ *m*, *m* ≥ 2), and let *A*(*x*, *y*) = *A*(*y*, *x*) = 〈(*x*, *y*, 1), (*x*, *y*, 2),…, (*x*, *y*, *m*)〉. Then we obtain an *m*-polar fuzzy set *A* : *X* → [0,1]^*m*^, which is a multivalued similarity relation.
2. Consider a set *X* consisting of *n* people *x*_1_, *x*_2_,…, *x*_*n*_  (*n* ≥ 2) in a social network. For *x*, *y* ∈ *X*, we use (*x*, *y*, *j*) to denote the degree of connection between *x* and *y* in *j*th aspect (1 ≤ *j* ≤ *m*, *m* ≥ 2), and let *A*(*x*, *y*) = *A*(*y*, *x*) = 〈(*x*, *y*, 1), (*x*, *y*, 2),…, (*x*, *y*, *m*)〉. Then we obtain an *m*-polar fuzzy set *A* : *X* → [0,1]^*m*^, which is a multivalued social graph (or multivalued social network) model.

## 3. Conclusion

In this note, we show that the enthusiastically studied notion of bipolar fuzzy set is actually a synonym of a [0,1]^2^-set (we call it 2-polar fuzzy set), and thus we highlight the notion of *m*-polar fuzzy set (actually a [0,1]^*m*^-set, *m* ≥ 2). The *m*-polar fuzzy sets not only have real backgrounds (e.g., “multipolar information” exists) but also have applications in both theory and real world problems (which have been illustrated by examples).

## Figures and Tables

**Figure 2:**